# Vacuum-Filtered MXene/Carbon Nanotube Composite Films for Li-Ion Capacitors

**DOI:** 10.1021/acsomega.5c05174

**Published:** 2025-08-04

**Authors:** Haojie Fei, Nikhitha Joseph, Elif Vargun, Matej Micusik, Petr Sáha

**Affiliations:** † Centre of Polymer Systems, Tomas Bata University in Zlín, Trida Tomase Bati 5678, 760 01 Zlín, Czech Republic; ‡ Chemistry Department, Faculty of Science, 229199 Muğla Sıtkı Koçman University, Kotekli, 48000 Muğla, Turkey; § Polymer Institute, 371075 Slovak Academy of Sciences, Dúbravská cesta 9, 845 41 Bratislava, Slovakia; ∥ University Institute, Tomas Bata University in Zlín, Nad Ovčírnou 3685, 760 01 Zlín, Czech Republic

## Abstract

MXene has garnered significant attention for its applications in electrochemical energy storage devices, such as supercapacitors and Li-ion capacitors, owing to its high electrical conductivity and relatively high capacitance/capacity in both aqueous and organic electrolytes. Utilizing its two-dimensional (2D) structure, this study prepared vacuum-filtered MXene/carbon nanotube (MXene/CNT) composite films for Li-ion capacitors. The incorporation of CNTs plays a critical role in mitigating the restacking of MXene flakes and enhancing the structural integrity of the films. The MXene/CNT films were first characterized by using various physicochemical methods and evaluated in electrochemical half-cells. A Li-ion capacitor was subsequently fabricated by using the MXene/CNT-12% film as the negative electrode and mesoporous carbon as the positive electrode. The fabricated Li-ion capacitor demonstrates a specific capacitance of 26 F g^–1^, an energy density of 40.2 Wh kg^–1^, and a power density of 375 W kg^–1^ at a current density of 0.5 A g^–1^. However, the electrochemical performance of the device is still limited by the layer-by-layer architecture of the MXene-based films, which hinders the efficient transport of electrolyte ions vertically through the layers.

## Graphical abstract

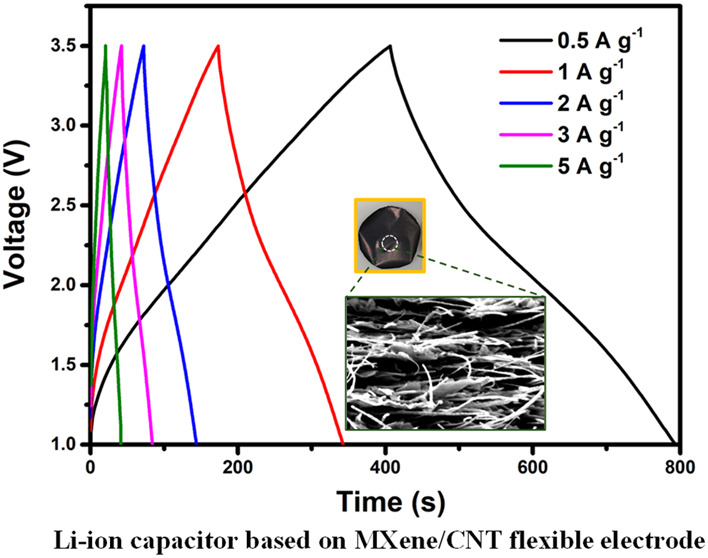

## 1 Introduction

MXenes are a class of two-dimensional (2D) transition metal carbides/nitrides with the general formula M_*n*+1_X*_n_* T_*x*_, where M denotes a transition metal, X signifies carbon and/or nitrogen, *n* is an integer ranging from 1 to 4, and T indicates the surface functional groups, with *x* representing the quantity of these functional groups.^1,2^ The most widely studied MXene, Ti_3_C_2_T_*x*_, is synthesized by selectively etching aluminum layers from the MAX phase precursor using hydrofluoric acid (HF) or fluoride salts. This process leaves the Ti_3_C_2_T_*x*_ surface terminated with hydrophilic functional groups (−OH, =O, −F).^3^ Ti_3_C_2_T_*x*_ demonstrates exceptional electrical conductivity (∼6600 S cm^–1^), substantial specific capacitances, and hydrophilic characteristics, indicating significant potential for applications in electrochemical storage systems.^4–6^

Ti_3_C_2_T_*x*_ has an outstandingly high specific capacitance (∼300 F g^–1^) in the H_2_SO_4_ electrolyte due to proton-induced pseudocapacitance.^7^ It has a low hydrogen evaluation potential (−1.1 V vs Hg/Hg_2_SO_4_), significantly increases the cell voltage of the asymmetrical supercapacitor combined with polyaniline and RuO_2_ positive electrodes, and exhibits superior cycling stability.^8,9^ However, it is still challenging in floating testing, and an initial capacitance drop occurs during the early constant potential steps. The extended voltages of these supercapacitors are still lower than those using organic electrolytes.^10^ Moreover, these aqueous supercapacitors require current collectors with high corrosion resistance and a low hydrogen evolution potential in negative electrodes, which are denser and costlier than the aluminum foils used in organic systems. Therefore, this kind of asymmetrical supercapacitor is still inferior to organic electrolyte systems. Interestingly, Ti_3_C_2_T_*x*_ also exhibits considerable specific capacity in the organic electrolytes utilized in Li-ion batteries, attributed to Li-ion (de)­intercalation.^11^ This material has been investigated for its application as the high-rate negative electrode in Li-ion batteries and Li-ion capacitors.^12,13^ It exhibits an elevated energy density and power density.

The 2D structure of Ti_3_C_2_T_*x*_, along with its hydrophilic properties, facilitates the straightforward preparation of a flexible Ti_3_C_2_T_*x*_ film through vacuum filtration.^14^ This film shows significant potential for the application of flexible energy storage devices in wearable technology.^15,16^ The restacking of 2D Ti_3_C_2_T_*x*_ within the film may impede electrolyte ion transport through the electrode, resulting in reduced specific capacitance and low-rate capability. This phenomenon also occurs in flexible electrodes made from reduced graphene oxide (RGO).^17^ Taking the experience of preparing RGO flexible electrodes, intercalated composite Ti_3_C_2_T_*x*_ films and Ti_3_C_2_T_*x*_ hydrogel films have been prepared, demonstrating an improved electrochemical performance.^18–20^

Herein, we prepared acid-treated multiwall carbon nanotube (CNT)-incorporated Ti_3_C_2_T_*x*_ (MXene/CNT) films through vacuum filtration for using them as the negative electrodes in Li-ion capacitors. The influence of the CNT content on the morphology and electrochemical performance of MXene films was investigated. A reduced graphene oxide/mesoporous carbon (Graphene/Meso-carbon) composite was synthesized for use as the positive electrode. Prior to fabrication of a Li-ion capacitor, the capacities of the positive and negative electrodes were aligned across different current densities. The performance of the fabricated Li-ion capacitors demonstrates the applicability of MXene films in these devices.

## 2 Materials and Methods

### 2.1 Materials

Ti_3_AlC_2_ MAX was received from Polymer Institute, Slovak Academy of Sciences Bratislava, Slovakia, and originally from Materials Research Center, Kyiv, Ukraine. Lithium fluoride (LiF), hydrochloric acid (HCl), multiwalled CNTs (50–90 nm diameter), *N*-methyl-2-pyrrolidone (NMP, 99.5%, anhydrous), poly­(tetrafluoroethylene) (PTFE, 60 wt % dispersion in H_2_O), poly­(vinylidene fluoride) (PVDF, average *M*_w_ ∼ 530,000, pellets), and 1 M LiPF_6_ in ethylene carbonate (EC) and dimethyl carbonate (DMC) (1:1) electrolyte were purchased from Sigma-Aldrich, Czechia. Polycarbonate (PC) membranes (0.22 μm) and Durapore membranes (0.22 μm, hydrophilic PVDF) were purchased from Sigma-Aldrich. Celgard 2500 was purchased from Cambridge Energy Solutions, U.K.

### 2.2 Methods

#### 2.2.1 Preparation of MXene

Few-layer Ti_3_C_2_T_*x*_ MXene was synthesized through the etching of Ti_3_AlC_2_ MAX in a LiF and HCl solution.^21^ In a typical procedure, 1 g of Ti_3_AlC_2_ was gradually introduced into a 20 mL solution of 37% HCl containing 1.6 g of LiF. The mixture was stirred continuously at 37 °C for a duration of 24 h. Multilayer Ti_3_C_2_T_*x*_ was obtained via centrifugation and washed with deionized water until the pH of the supernatant was approximately 7. Ti_3_C_2_T_*x*_ was redispersed in deionized water and subjected to bath sonication for a duration of 15 min. The procedure was conducted four times, utilizing centrifugation at 3500 rpm for 1 h and sonication for 15 min to yield a colloidal solution of few-layer Ti_3_C_2_T_*x*_, along with a black supernatant. Additionally, Ti_3_C_2_T_*x*_ powders were obtained through lyophilization of the colloidal solution.

#### 2.2.2 Synthesis of Acid-Treated CNTs

Multiwalled CNTs were subjected to treatment with a HNO_3_/H_2_SO_4_ mixture (1:3) while being mechanically stirred for 1 h. The mixture underwent sonication for 5 min and was subsequently stirred at 60 °C for 6 h, with two additional sonication intervals included. The acid-treated CNTs underwent multiple washings and dialysis with deionized water.

#### 2.2.3 Preparation of MXene/CNT Films

MXene/CNT films were prepared by vacuum-filtering an aqueous mixture of few-layer Ti_3_C_2_T_*x*_ and acid-treated CNTs via PC membranes. The obtained films were dried at 50 °C for 3 h. MXene films with various amounts of CNTs (4, 8, and 12 wt %) were prepared. For comparison, pure MXene films were also prepared without adding CNTs. Different filter membranes were also used to optimize the preparation.

#### 2.2.4 Material Characterization

Morphological characterization was conducted with a Nova Nano (SEM 450) scanning electron microscope (SEM) manufactured by the FEI Company. X-ray diffraction (XRD) patterns were recorded on a Rigaku MiniFlex 600 diffractometer using a CoKα (λ = 1.7903 Å) radiation source at an operating voltage of 40 kV and a scan rate of 10° min^–1^.

#### 2.2.5 Electrochemical Measurements

The obtained Ti_3_C_2_T_*x*_ powders were subsequently mixed with carbon black (super P) and a PVDF binder in a weight ratio of 80:10:10 in NMP. The slurry was then cast onto a copper foil current collector, from which 14 mm diameter disks were punched. Graphene/Meso-carbon (a specific surface area of 870.4 m^2^ g^–1^, a mean pore diameter of 7.7 nm, Brunauer–Emmett–Teller (BET) method) was used as the positive electrode.^22^ The powders were mixed with carbon black and a PTFE dispersion (80:10:10) in ethanol. The paste was cold-rolled, and 14 mm diameter disks were punched. The mass loading was then adjusted, depending on the mass of the negative material. Finally, the electrodes were dried at 120 °C in a vacuum oven for 12 h. Half-cells and full-cells were fabricated in CR2032 coin cells. Glass fiber separators (Whatman, GF/A) and 1 M LiPF_6_ in EC/DMC were used. For the full-cell assembly, the MXene/CNT negative electrode was first precycled in the half-cell but without prelithiation. Galvanostatic charge/discharge cycling and cyclic voltammetry (CV) tests were performed by using a battery cycler (BCS810, Biologic).

## 3 Results and Discussion

### 3.1 SEM of MAX, MXene, and CNTs

The Ti_3_AlC_2_ MAX phase crystallizes in a hexagonal layered structure, characterized by alternating covalent Ti–C layers and metallic Ti–Al layers. This layered architecture is evident in the SEM image (Figure 1a), which reveals platelike/lamellar grains with lateral dimensions of 5–7 μm, consistent with the anisotropic growth typical of MAX phases. Selective etching of the Al layers converts Ti_3_AlC_2_ into few-layer Ti_3_C_2_T*_x_* MXene, as confirmed by Figure 1b. The transition from bulky MAX grains to thin and delaminated MXene flakes validates successful Al removal. The dark contrast observed in the SEM image for MXene arises from its ultrahigh electrical conductivity, which minimizes electron accumulation during imaging. Figure 1c demonstrates that acid-treated CNTs retain their structural integrity and high aspect ratio after acidic functionalization. These CNTs act as conductive intercalation fibers within MXene composite films, enhancing mechanical robustness while preserving electrical connectivity, which is critical for flexible electrodes.

**Figure 1:**
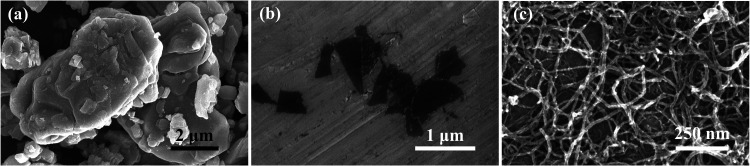
SEM image of (a) Ti_3_AlC_2_ MAX, (b) few-layer Ti_3_C_2_T_*x*_ MXene on Al foil, and (c) acid-treated CNTs.

### 3.2 Vacuum Filtration of MXene-Based Films

Figure 2a illustrates the vacuum filtration process used to fabricate flexible MXene and MXene/CNT composite electrodes. The inset image in Figure 2a highlights the stable aqueous dispersion of the acid-treated CNTs and MXene flakes. The uniform distribution of CNTs within the MXene matrix is crucial for enhancing the mechanical integrity and electrical conductivity of the composite films. This stability arises from their negative surface charges, which prevent aggregation through electrostatic repulsion.^23,24^ Vacuum filtration was selected for its ability to produce uniform, dense films with a controlled thickness, which is essential for electrochemical applications. The filtration membrane is crucial for the effective preparation of the MXene films. In contrast to GO or RGO, MXene exhibits an absence of wrinkles, resulting in a reduced stretchability of MXene films. The mechanical neutral plane must be positioned within MXene during the filtration and drying process, necessitating a thin and soft filtration membrane. However, Celgard 2500 exhibits excessive softness, resulting in deformation during the filtration process, as shown in Figure 2b. A flat substance is necessary at the base of the Buchner funnel. To detach the MXene films from the filtration membrane, the mechanical neutral plane should also be located within MXene, in contrast to the filtration preparation of GO or RGO films, where the mechanical neutral plane may reside in the filtration membrane. The size of the pores in the filtration membrane is significant. MXene flakes should not be confined within the pores. The hydrophilicity of the filtration membrane is also crucial. The highly hydrophilic surface of the membrane enhances its interaction with the MXene flakes, complicating the peeling process. The MXene film adheres to the hydrophilic PVDF membrane following the filtration process, as shown in Figure 2b.

**Figure 2:**
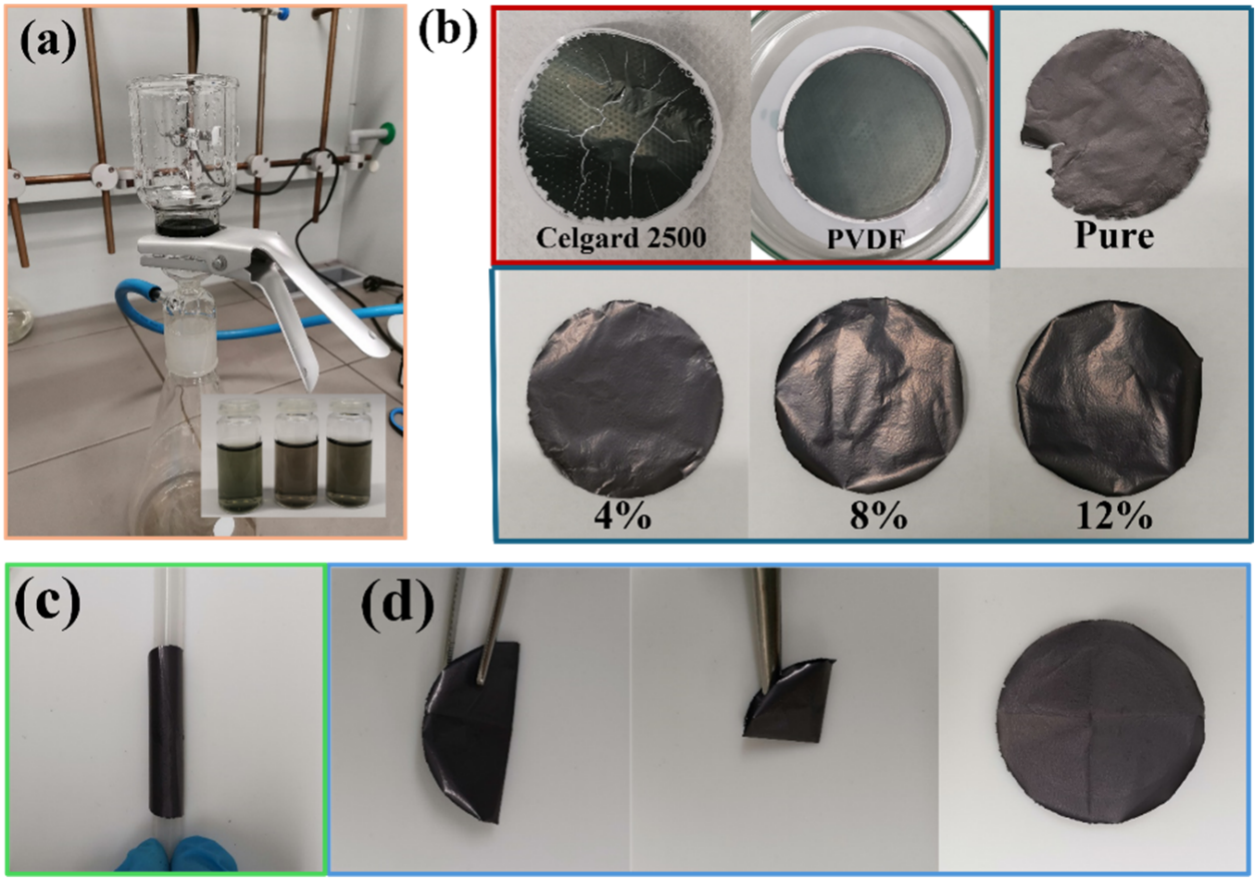
MXene and composite film preparation. (a) Vacuum filtration setup and stable MXene and CNT colloids (inset); (b) digital images of pure MXene films vacuum-filtered by Celgard 2500, PVDF, and polycarbonate membranes, respectively, and composite films with varying CNT contents using polycarbonate membranes; (c) roll of the MXene composite film; and (d) folding and unfolding of the MXene composite film.

A PC membrane with a pore size of 0.22 μm served as the filtration substrate, chosen for its softness and smooth surface, which facilitates easy peeling of freestanding films postdrying. The pure MXene and MXene/CNT composite films were successfully prepared and they exhibited exceptional flexibility, as shown in Figure 2b. The MXene/CNT-8% composite demonstrates remarkable durability under mechanical stress; it can be tightly rolled (Figure 2c), folded, and unfolded (Figure 2d) without cracking or delamination. This robustness is attributed to the CNTs acting as reinforcing agents. Excessive CNT content, however, leads to an increased brittleness. Hence, the content of CNTs was restricted to 12 wt %. The mass loading values for MXene, MXene/CNT-4%, MXene/CNT-8%, and MXene/CNT-12% are 1.43, 1.56, 1.69, and 1.95 mg cm^–2^, respectively.

### 3.3 XRD Analysis

Figure 3 presents the XRD patterns of raw MAX, MXene powders, pure MXene films, and MXene/CNT films. A series of characteristic peaks are observed at *2*θ = 9.20, 18.80, 33.69, 35.65, 38.51, 41.46, 48.15, and 56.15°, corresponding to the (002), (004), (101), (103), (104), (105), (107), and (109) planes of the Ti_3_AlC_2_ MAX phase. The presence of a distinct (002) peak at 6.2° in the XRD patterns of a pure MXene film confirms the successful removal of aluminum (Al) atoms from Ti_3_AlC_2_. Few-layer MXene was obtained, as the peak position is affected by the distances between MXene layers, with smaller values indicating a larger c-lattice.^25^ The heightened intensity of the distinct (002) peak signifies a decrease in the restacking of MXene, thereby demonstrating the effective separation of MXene flakes by CNTs. The peak at an angle of 26.1° is observed in MXene/CNT films, with the intensity increasing as the quantity of CNTs is increased. The observed peak aligns with the (002) reflection of sp^2^ carbon, indicating that the acid treatment had minimal impact on the quality and crystallinity of CNTs.^26^ It is also confirmed by the Raman spectrum of the acid-treated CNT (Figure S1), which displays a low I_D_/I_G_ ratio of 0.59 and a high-intensity G′ band.

**Figure 3:**
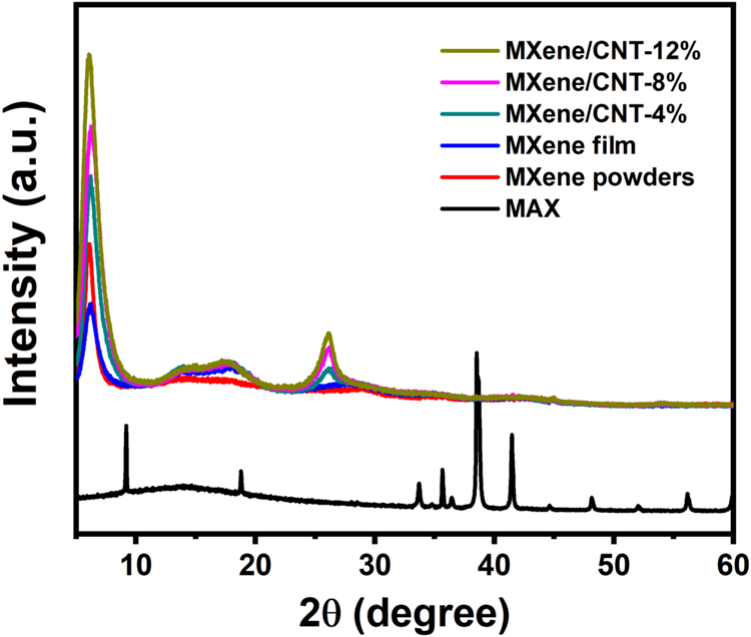
XRD patterns of raw MAX, MXene powders, the pure MXene film, and MXene composite films with different amounts of acid-treated CNTs.

### 3.4 SEM of MXene-Based Films

Figure 4 illustrates the surface and cross-sectional morphologies of MXene and MXene/CNT films. The pure MXene film displays a smooth, wrinkle-free surface (Figure 4a), suggesting significant restacking of MXene flakes after filtration and drying. CNTs are observed on the surfaces of MXene/CNT-8% and MXene/CNT-12% films (Figure 4c,d). They are intertwined and, meanwhile, overlap with the MXene flakes. A rough surface is elevated. The contact angles of 1 M LiPF_6_ in EC/DMC on various MXene-based films were measured. The results are shown in Figure S2. Because of the increased surface roughness of the MXene-based films and the polar functional groups on CNTs, the contact angle decreases as the CNT concentration rises. However, no significant porosity is observed in this microscale (Figure 4d), potentially hindering ion transport pathways and diminishing rate capability in electrochemical devices.

**Figure 4:**
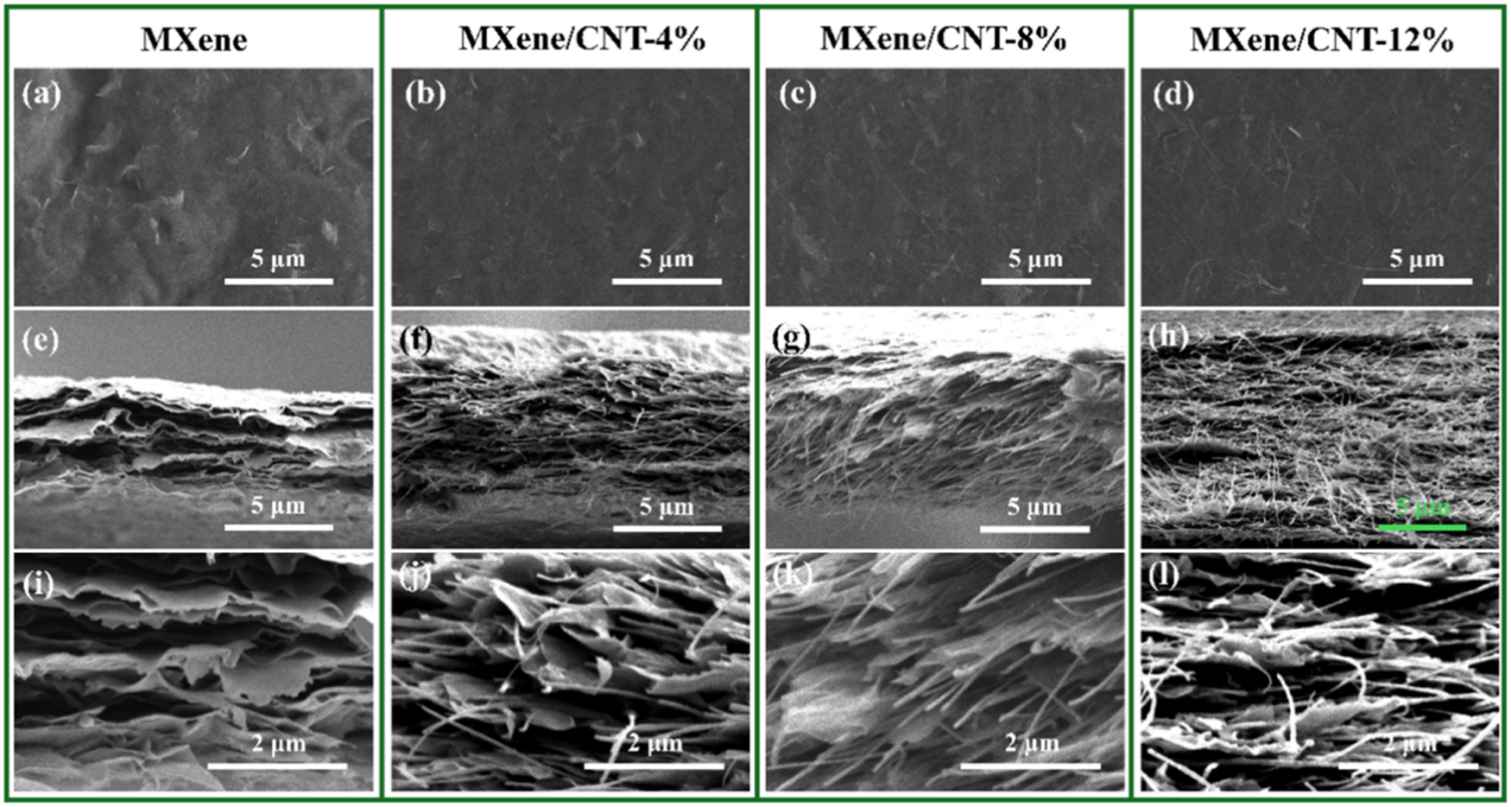
Surface top view and cross-sectional Figure 4. SEM images of (a–d) surface topography, (e–h) low-magnification cross-sections, and (i–l) high-magnification cross-sections for pure MXene and MXene/CNT composite films morphology of MXene and MXene/CNT films.

Figure 4e,i illustrates the cross-sectional morphology of MXene and MXene composite films at both low and high magnifications. The pure MXene film exhibits a tightly stacked, lamellar structure resulting from the van der Waals-driven restacking of MXene flakes. CNTs serve as spacers between MXene layers (Figure 4f–h,j–l), reducing restacking and facilitating the formation of nano/microscale voids for electrolyte infiltration. The thickness increases with an increase in the CNT content. The electrical conductivity of the film decreases from 2350 S cm^–1^ for the pure MXene film to 1232 S cm^–1^ for MXene/CNT-12%, attributed to the low electrical conductivity of CNTs and the increased spacing. Furthermore, the cross-sectional SEM-EDX elemental mapping of epoxy-embedded MXene/CNT-12% was conducted and is shown in Figure S3, highlighting the distribution of Ti, O, and C in the MXene/CNT composite film.

### 3.5 Electrochemical Performance of MXene Powders

Before electrochemical evaluation of the MXene and MXene/CNT composite films, the pristine MXene powder was analyzed to assess its baseline performance. Figure 5a presents the cyclic voltammetry (CV) curves of the MXene powder-based electrode in a half-cell configuration at varying potential windows. Within the range of 1.1–3.0 V vs Li/Li^+^, where no solid electrolyte interphase (SEI) forms, the electrode delivered a modest specific capacity of 52 mAh g^–1^ at a scan rate of 0.1 mV s^–1^. Expanding the electrochemical window to 0.5 V vs Li/Li^+^ resulted in an enhanced capacity of 94 mAh g^–1^, attributed to the formation of a thin SEI layer while maintaining high-rate capability. Further lowering the potential to 0.05 V vs Li/Li^+^ increased the capacity to 156 mAh g^–1^. However, this deeper lithiation likely promotes a thicker SEI layer and introduces contributions from carbon black additives via Li-ion intercalation/deintercalation processes, complicating the capacity interpretation.^12^

**Figure 5:**
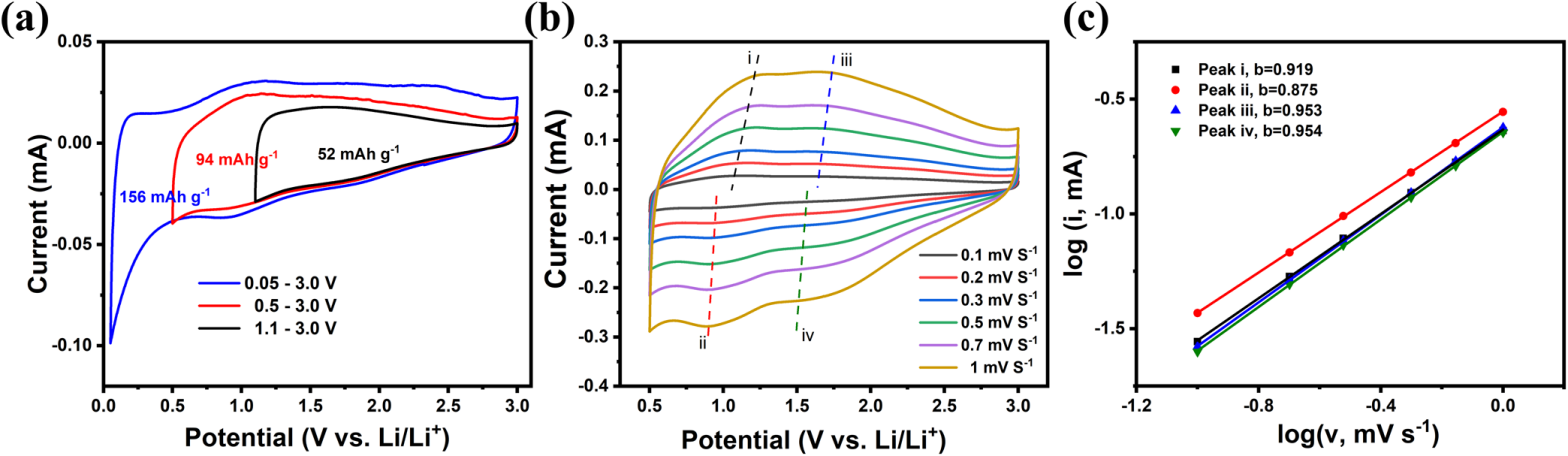
(a) CVs of MXene powders at various potential windows; (b) CVs of MXene powders at various scan rates; and (c) determination of the corresponding *b*-value using the peak current to scan rate.

The potential window was kept at 0.5–3.0 V vs Li/Li^+^ to balance the capacity and rate performance. To investigate the high-rate capability of the MXene powder-based electrode, its reaction kinetics were analyzed using CV curves at varying scan rates (Figure 5b). The CV profiles retained similar shapes across scan rates, with peak currents increasing proportionally as scan rates rose. The peak current (*i*) has a relationship with the scan rate (*v*) by the following equation

$$i=av^{b}$$

Both *a* and *b* are adjustable parameters. In particular, *b* = 0.5 represents a diffusion-controlled faradaic intercalation process, while *b* = 1 indicates a capacitive behavior via a surface faradaic redox reaction.^27^ From the slopes of the log­(*i*) vs log­(*v*) plot (Figure 5c), the calculated *b*-values were 0.919 and 0.953 for anodic peaks (i) and (iii) and 0.875 and 0.954 for cathodic peaks (ii) and (iv), respectively. These results indicate that the capacity is predominantly derived from capacitive and pseudocapacitive contributions.

### 3.6 Electrochemical Performance of MXene-Based Films

The pure MXene and MXene/CNT films were then fabricated into the half-cell by using Li metal as the counter and reference electrodes. During the extensive restacking of MXene flakes in a pure MXene film, it exhibits minimal capacity. MXene/CNT-4% has a markedly low specific capacity, as illustrated in Figure 6a. Extending the rest period after cell assembly to allow the electrolyte to permeate the electrode can enhance the specific capacity. The incorporation of CNTs reduced the amount of restacking MXene flakes and increased the interstitial space within the electrode. It is beneficial for ion transport and regional electrolyte storage. MXene/CNT-12% exhibits a moderate specific capacity of 54 mAh g^–1^ at a current density of 0.1 A g^–1^ relative to the overall mass of the electrode (Figure 6b). The reaction kinetics were examined by CV curves at different scan rates (Figure 6c). In comparison to the CV curves of MXene powders depicted in Figure 5b, the redox peaks in the CVs of MXene/CNT-12% at lower potentials are not pronounced due to the sluggish kinetics of the faradaic intercalation process. However, the computed *b*-value indicates a heightened contribution from faradaic intercalation (Figure 6d).

**Figure 6:**
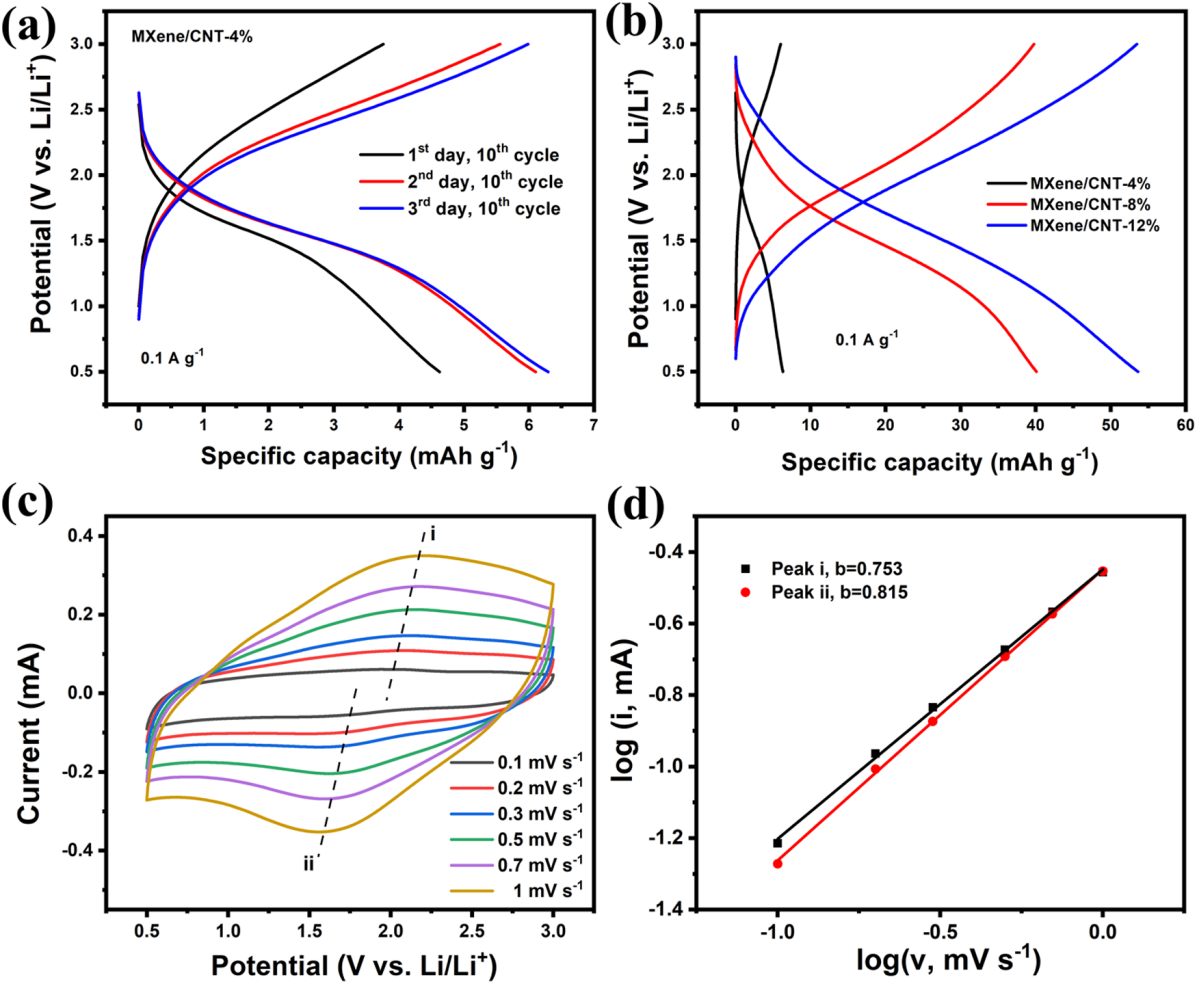
(a) Charge/discharge curves of the MXene/CNT-4% half-cell at different times after assembly; (b) charge/discharge curves of MXene composite films with various amounts of CNTs; (c) CVs of MXene/CNT-12%; and (d) the corresponding *b*-value found using the peak current relationship to scan rate.

### 3.7 Limited Ion Transport in the MXene-Based Films

Due to 2D few-layer MXene, the pure MXene film exhibits significant stacking, as illustrated in Figure 7a. This dense stacking does not allow the electrolyte to permeate the film. During charge–discharge cycles, ion transport occurs primarily through the gaps between MXene layers at the cross-sectional edges of the film. However, this pathway is quickly hindered by the restacking of the MXene flakes. Crucially, the high aspect ratio (radius/thickness ≈ 1167) of the pure MXene film imposes a major limitation; in the central region of the film, ions must traverse vertically through the MXene layers. Given the limited surface porosity (Figure 4a), this severely restricts ion accessibility, explaining the negligible capacity observed in the half-cell. Introducing CNTs as spacers between MXene flakes reduces stacking and enhances the porosity, as shown in Figure 7b. This modification facilitates faster ion diffusion through the interlayer gaps. Nevertheless, the long diffusion distance, resulting from the high aspect ratio (538), still necessitates vertical ion transport in the central region of the films. While CNT intercalation improves ion kinetics at the edges, this benefit diminishes in larger electrodes where bulk ion accessibility becomes critical. Thus, despite enhanced electrolyte permeation, fully exploiting the theoretical capacity of MXene and achieving a high-rate performance remains challenging. This explains that MXene/CNT-12% could not achieve a comparable specific capacity to the MXene powders.

**Figure 7:**
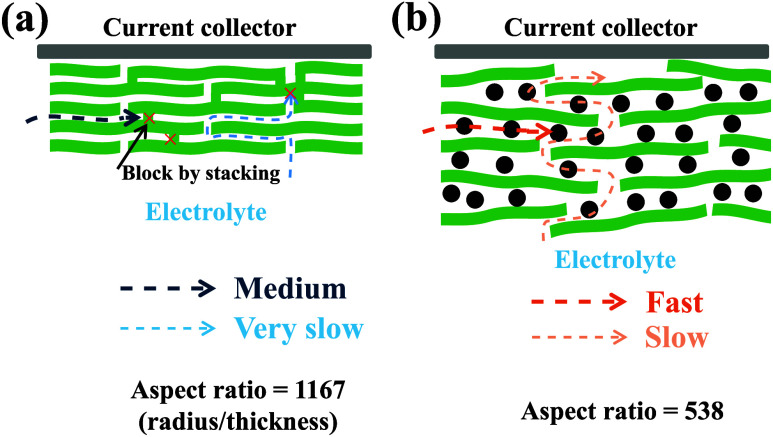
Ion transport pathway in (a) MXene and (b) MXene/CNT-8% films.

### 3.8 Fabrication of the Li-Ion Capacitor and Its Electrochemical Performance

Graphene/Meso-carbon served as the positive electrode. The structure is illustrated in the SEM and TEM images presented in Figure S4. Following the etching of the silica nanoparticles, mesoporous carbon derived from polyaniline is attached to the graphene oxide. The mesoporous carbon exhibits a uniform pore size of approximately 7 nm, attributed to the hard template method (Figure S4b). The Raman spectra of Graphene/Meso-Carbon, as illustrated in Figure S5, exhibit a low *I*_D_/*I*_G_ ratio of 0.99, indicating an ordered structure resulting from a high carbonization temperature of 900 °C.

To fabricate the Li-ion capacitor (full-cell), the mass ratio between the positive electrode (Graphene/Meso-carbon) and the negative electrode (MXene/CNT-12%) was determined using the charge-balancing principle, where the capacity of the positive electrode (*Q*_*+*_) must equal that of the negative electrode (*Q*_*–*_). Figure 8a shows the CV profiles of the MXene/CNT-12% and Graphene/Meso-carbon electrodes. The working potential windows for MXene/CNT-12% and Graphene/Meso-carbon are 0.5–3.0 and 3.0–4.2 V vs Li/Li^+^, respectively, enabling a maximum full-cell voltage of 3.7 V. The cell voltage of the Li-ion capacitor was subsequently determined to be 3.5 V, owing to the largely polarized currents observed at 4.2 and 0.5 V vs Li/Li^+^ for the positive and negative electrodes, respectively. To account for the current-density-dependent variation in the specific capacity, rate performance tests were conducted via galvanostatic charge/discharge measurements. The specific capacity dependence on the current density for the positive and negative electrodes is shown in Figure 8b,c, respectively. Based on these results, the mass ratio of the positive to the negative electrode was optimized to 2, ensuring balanced capacities (*Q*_*+*_ = *Q*_*–*_) across most tested current densities.

**Figure 8:**
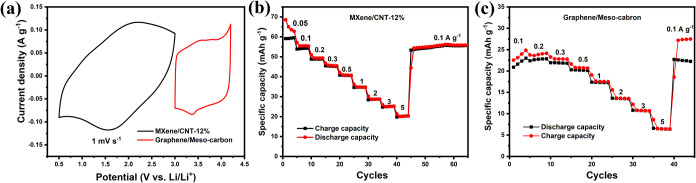
(a) CVs of MXene/CNT-12% and Graphene/Meso-carbon. Specific charge/discharge capacity dependency on current densities of (b) MXene/CNT-12% and (c) Graphene/Meso-carbon.

Figure 9a,b presents the charge/discharge profiles of the fabricated Li-ion capacitors at various current densities (based on the total mass of MXene/CNT-12%). The plots are shown as potential (*E*) vs time (typical for capacitors and supercapacitors) and potential (*E*) vs capacity (typical for batteries). Owing to the dominant capacitive and pseudocapacitive energy storage mechanisms of both positive and negative electrodes, the charge/discharge curves exhibit a linear behavior, with potential increasing and decreasing proportionally over time and capacity. The specific capacitance of the device, calculated based on the total mass of both electrodes, is 26 F g^–1^, and the specific capacity of the negative electrode is 53 mAh g^–1^ at 0.5 A g^–1^. At this current density, it demonstrated an energy density of 40.2 Wh kg^–1^ and a power density of 375 W kg^–1^ based on the total mass of two electrodes. The Ragone plot of this Li-ion capacitor is presented in Figure S6. The energy density constraint of the Li-ion capacitor arises from the low specific capacity of initial Ti_3_C_2_T_*x*_ (MXene) and the lower specific capacity of MXene-based films resulting from MXene stacking. The intercalation of CNTs could reduce the restacking. However, the layer-by-layer architecture of MXene-based films obstructs the passage of electrolyte ions through the layers. In comparison to the aqueous electrolytes, the Li-ion organic electrolytes exhibit a higher viscosity and larger ion sizes, resulting in a greater loss of specific capacity. Creating porosity through the MXene layers without damaging the film structure is essential for the MXene-based films used in Li-ion capacitors.^28,29^ However, this may compromise mechanical properties and flexibility, offsetting the benefits of the MXene films’ high volumetric energy density.

**Figure 9:**
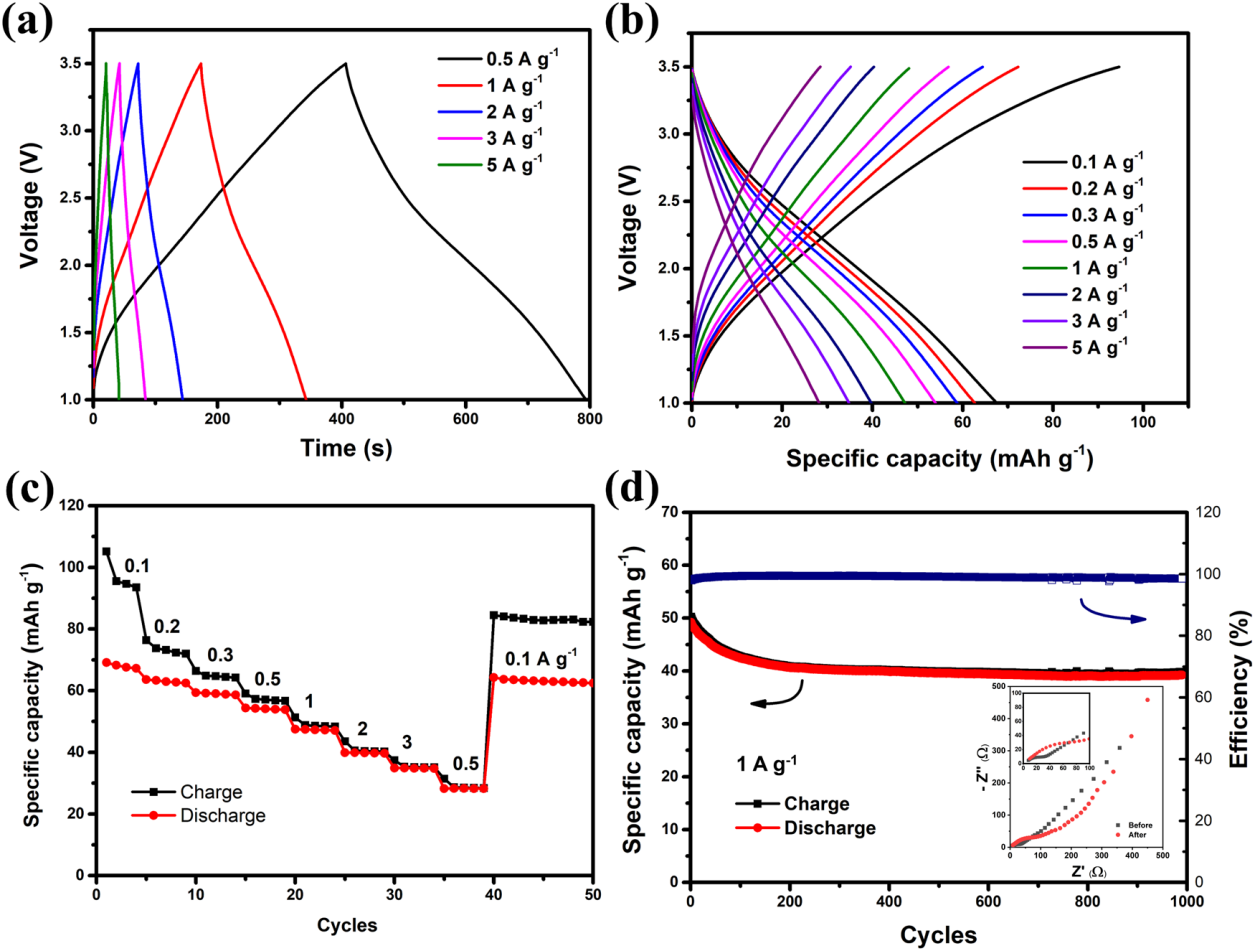
Charge/discharge curves of the Graphene/Meso-carbon//MXene/CNT-12% full-cell based on (a) *E* vs time and (b) *E* vs capacity; (c) dependency of the specific capacity on current densities; and (d) cycling stability and Nyquist plots before and after 1000 cycles (inset).

The dependence of the specific capacity on the current density is illustrated in Figure 9c. A low Coulombic efficiency is observed at low current densities (0.1–0.5 A g^–1^), attributed to side reactions of porous carbon at elevated potentials in the Li-ion organic electrolytes.^30,31^ Therefore, it is recommended that this Li-ion capacitor be operated at current densities above 0.5 A g^–1^. Figure 9d displays the cycling stability of the Li-ion capacitor at 1 A g^–1^ over 1000 cycles. A noticeable capacity decline occurs during the initial 200 cycles with approximately 20% of the initial capacity lost. Subsequently, the capacity stabilizes, retaining 76% of the initial capacity by the end of the test. Similarly, the Coulombic efficiency is relatively low in the early stages but stabilizes to nearly 100% in later cycles. The Nyquist plot of the impedance analysis is shown in Figure 9d (inset). The charge transfer resistance increases after 1000 cycles due to the degradation of both positive and negative electrodes.

## 4 Conclusions

A Li-ion capacitor was fabricated using MXene/CNT-12% and Graphene/Meso-carbon. The specific capacitance of the device, determined from the total mass of both electrodes, is 26 F g^–1^ at a rate of 0.5 A g^–1^. At this current density, it demonstrates an energy density of 40.2 Wh kg^–1^ and a power density of 375 W kg^–1^. The Li-ion capacitor retained 76% of the initial capacity at 1 A g^–1^ over 1000 cycles. However, the layer-by-layer structure of MXene-based films, even the optimized film MXene/CNT-12%, still hinders the electrolyte ion transport across the layers. The capacity degradation observed in such systems can be attributed to two primary factors: (1) the inherently low Coulombic efficiency of porous carbon electrodes at low current densities and (2) the absence of an excess lithium source to compensate for irreversible lithium loss during cycling.

## Supplementary Material



## Notes

Raman spectrum of the acid-treated CNT; contact angles of 1 M LiPF6 in EC/DMC on (a) MXene, (b) MXene/CNT-4%, (c) MXene/CNT-8%, and (d) MXene/CNT-12%; cross-sectional SEM-EDX elemental mapping of epoxy-embedded MXene/CNT-12%, highlighting the distribution of Ti, O, and C; SEM and TEM images of Graphene/Meso-carbon; Raman spectrum of Graphene/Meso-carbon; and Ragone plot of the assembled Li-ion capacitor (PDF)
