# Supplementary material for: Vacuum-Filtered MXene/Carbon Nanotube Composite Films for Li-Ion Capacitors
Source: ACS Omega. 2025 Aug 4;10(32):36527–35. doi: 10.1021/acsomega.5c05174 (PMC12368816; doi:10.1021/acsomega.5c05174)
Supplement: Supplementary file 1 [file ao5c05174_si_001.pdf]

Supporting Information

Vacuum-Filtered MXene/Carbon Nanotube Composite Films for Li-Ion Capacitors

Haojie Fei[a, *], Nikhitha Joseph[a], Elif Vargun[b], Matej Micusik[c], Petr Sáha[d]

[a]Centre of Polymer Systems, Tomas Bata University in Zlín, Trida Tomase Bati, 5678, 760 01 Zlín, Czech Republic

[b]Chemistry Department, Faculty of Science, Muğla Sıtkı Koçman University, Kotekli, 48000 Muğla-Turkey

[c]Polymer Institute, Slovak Academy of Sciences, Dúbravská cesta 9, 845 41 Bratislava, Slovakia

[d]University Institute, Tomas Bata University in Zlín, Nad Ovčírnou 3685, 760 01 Zlín, Czech Republic

Material Characterization

Raman spectra were collected using a Thermo Scientific DXR Raman microscope (Thermo Fisher Scientific, Waltham, MA, USA), operating with a 532 nm laser line. The transmission electron microscope TEM images of Graphene/Meso-carbon was taken by FEI Titan Themis 60–300 (FEI, USA). The elemental analysis of MXene/CNT-12% was conducted using Nova Nano SEM 450 (FEI, USA), together with an energy-dispersive X-ray detector (EDX). The surface wettability of MXene-based films was evaluated by a SEE system (by Advex Instruments, Brno, Czech Republic).

The specific capacitance of Li-ion capacitor based on two electrodes (Ct) was calculated from the GCD curves at various current densities using the following formula:

$$C_t = I\Delta t/M\Delta V \quad (1)$$

$$\Delta V = V_{max} - V_{min} \quad (2)$$

where I is the discharge current (A), Δt stands for the discharge time (s), M is the total mass of two electrodes (g), and ΔV is the range of working voltage (V). It equals V_{max}-V_{min}, where V_{max} is the maximum voltage excluding IR_{drop} in the discharge curve, V_{min} is the cut-off voltage. Finally, the energy density (E, Wh/kg) and power density (P, W/kg) of each device were calculated using the provided equation:

$$E = I\Delta t(V_{max} + V_{min})/7.2M \quad (3)$$

$$P = 3600\,E/\Delta t \quad (4)$$

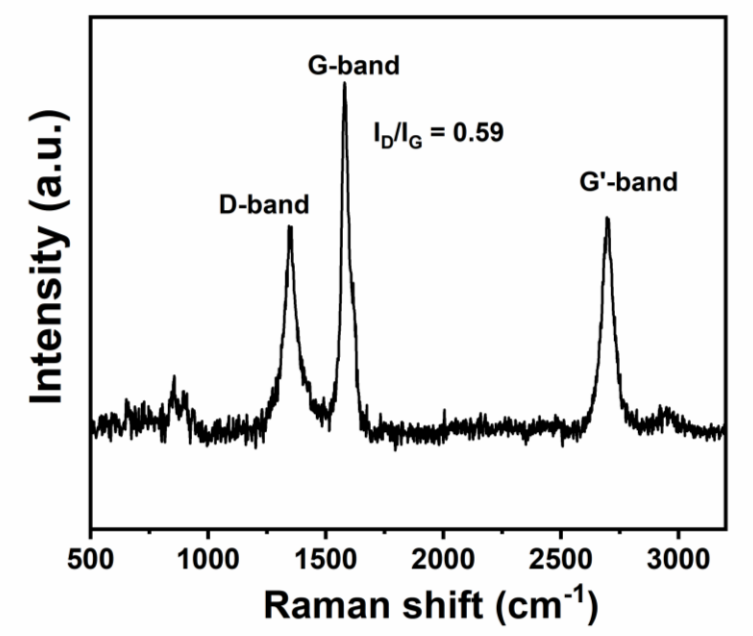

Figure S1. The Raman spectrum of acid-treated CNT

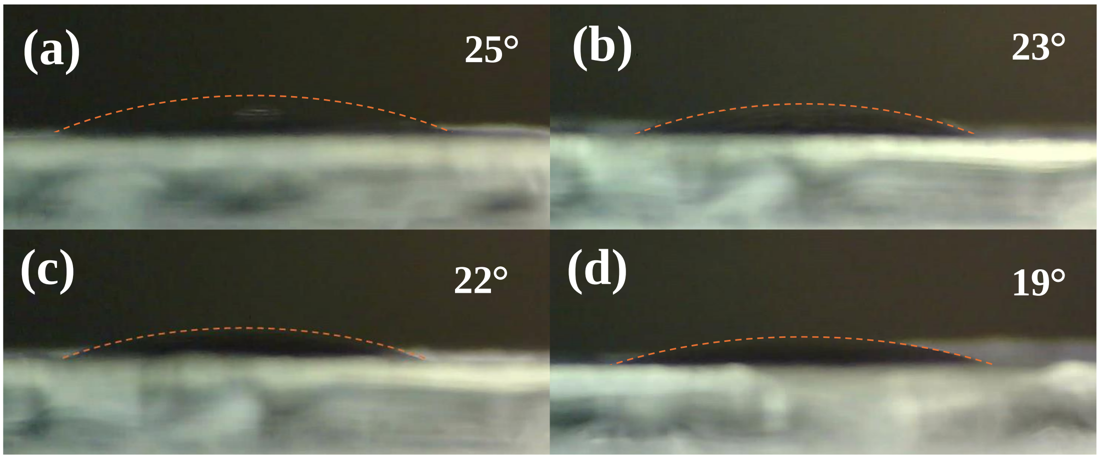

Fig. S2. The contact angles of 1M LiPF6 in EC/DMC on (a) MXene, (b) MXene/CNT-4%, (c) MXene/CNT-8%, and (d) MXene/CNT-12%

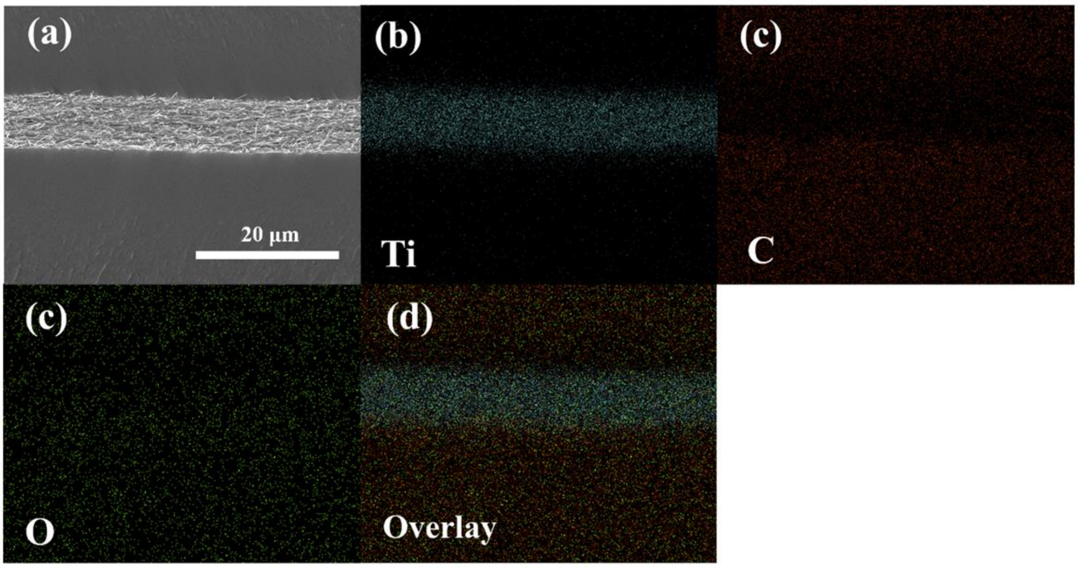

Figure S3. Cross-sectional SEM-EDX elemental mapping of an epoxy-embedded MXene/CNT-12%, highlighting the distribution of Ti, O, and C.

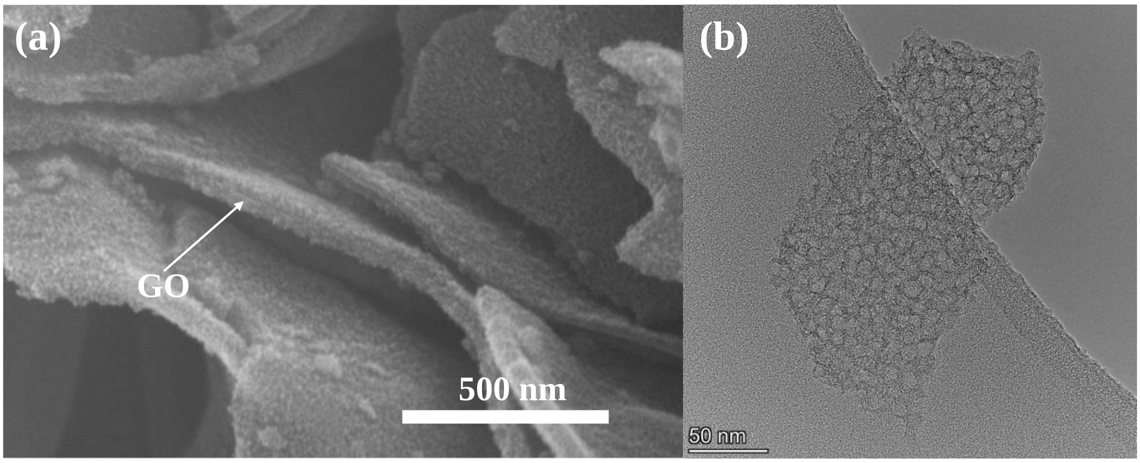

Figure S4. SEM and TEM images of Graphene/Meso-carbon.

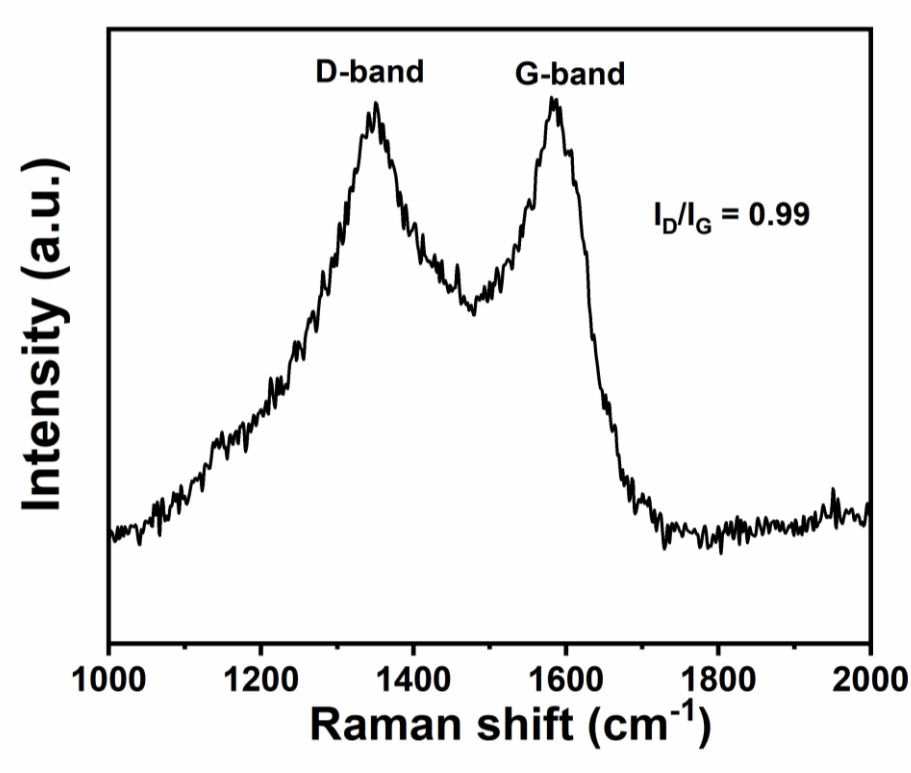

Fig. S5. The Raman spectrum of Graphene/Meso-carbon

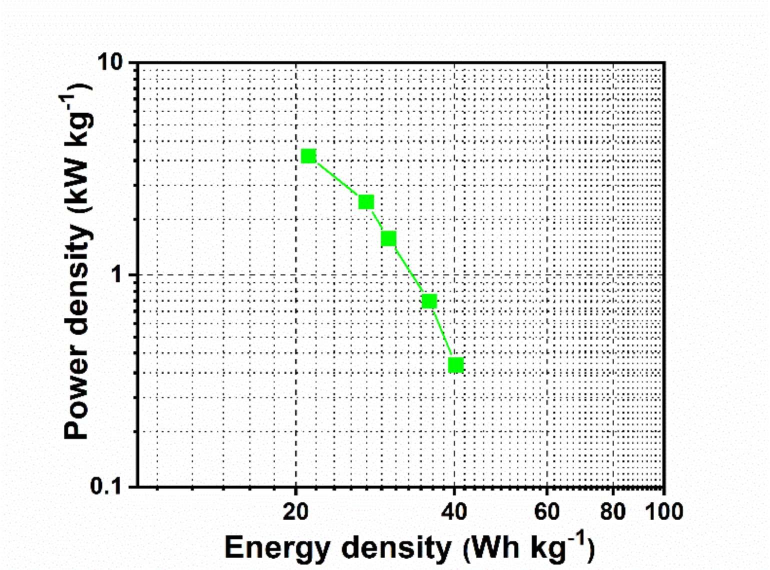

Fig. S6 Ragone plot of the assembled Li-ion capacitor
